# Vitamin D and Risk of Neuroimaging Abnormalities

**DOI:** 10.1371/journal.pone.0154896

**Published:** 2016-05-11

**Authors:** Thomas J. Littlejohns, Katarina Kos, William E. Henley, Iain A. Lang, Cedric Annweiler, Olivier Beauchet, Paulo H. M. Chaves, Bryan R. Kestenbaum, Lewis H. Kuller, Kenneth M. Langa, Oscar L. Lopez, David J. Llewellyn

**Affiliations:** 1 Clinical Trial Service Unit and Epidemiological Studies Unit, Nuffield Department of Population Health, University of Oxford, Oxford, United Kingdom; 2 University of Exeter Medical School, University of Exeter, Exeter, United Kingdom; 3 Department of Neuroscience, Geriatrics Division, Angers University Hospital, Angers, France; 4 Herbert Wertheim College of Medicine, Florida International University, Miami, United States of America; 5 Kidney Research Institute, Division of Nephrology, University of Washington, Seattle, United States of America; 6 Department of Epidemiology, University of Pittsburgh, Pittsburgh, United States of America; 7 Division of General Medicine, University of Michigan Health System, Ann Arbor, Michigan, United States of America; 8 Institute for Social Research, Institute of Gerontology and Institute for Healthcare Policy and Innovation, University of Michigan, Ann Arbor, Michigan, United States of America; 9 Veteran Affairs Center for Clinical Management Research, Ann Arbor, Michigan, United States of America; 10 Department of Neurology and Psychiatry Division of General Medicine, University of Pittsburgh, Pittsburgh, United States of America; University of Alabama at Birmingham, UNITED STATES

## Abstract

Vitamin D deficiency has been linked with an increased risk of incident all-cause dementia and Alzheimer’s disease. The aim of the current study was to explore the potential mechanisms underlying these associations by determining whether low vitamin D concentrations are associated with the development of incident cerebrovascular and neurodegenerative neuroimaging abnormalities. The population consisted of 1,658 participants aged ≥65 years from the US-based Cardiovascular Health Study who were free from prevalent cardiovascular disease, stroke and dementia at baseline in 1992–93. Serum 25-hydroxyvitamin D (25(OH)D) concentrations were determined by liquid chromatography-tandem mass spectrometry from blood samples collected at baseline. The first MRI scan was conducted between 1991–1994 and the second MRI scan was conducted between 1997–1999. Change in white matter grade, ventricular grade and presence of infarcts between MRI scan one and two were used to define neuroimaging abnormalities. During a mean follow-up of 5.0 years, serum 25(OH)D status was not significantly associated with the development of any neuroimaging abnormalities. Using logistic regression models, the multivariate adjusted odds ratios (95% confidence interval) for worsening white matter grade in participants who were severely 25(OH)D deficient (<25 nmol/L) and deficient (≥25–50 nmol/L) were 0.76 (0.35–1.66) and 1.09 (0.76–1.55) compared to participants with sufficient concentrations (≥50 nmol/L). The multivariate adjusted odds ratios for ventricular grade in participants who were severely 25(OH)D deficient and deficient were 0.49 (0.20–1.19) and 1.12 (0.79–1.59) compared to those sufficient. The multivariate adjusted odds ratios for incident infarcts in participants who were severely 25(OH)D deficient and deficient were 1.95 (0.84–4.54) and 0.73 (0.47–1.95) compared to those sufficient. Overall, serum vitamin D concentrations could not be shown to be associated with the development of cerebrovascular or neurodegenerative neuroimaging abnormalities in Cardiovascular Health Study participants.

## Introduction

Recent prospective studies have found that low serum vitamin D concentrations are associated with an increased risk of incident all-cause dementia [1], Alzheimer disease (AD) dementia [1,2] and non-AD dementias [3]. Neuroimaging techniques could provide an insight into the potential mechanisms underlying these associations. Cross-sectional studies have consistently found that lower vitamin D concentrations are associated with an increased risk of neuroimaging abnormalities [4–19]. These include cerebrovascular and neurodegenerative pathologies implicated in the development of dementia, such as white matter hyperintensities [4,10,12,13,15], enlarged ventricular volume [8], large vessel infarcts [4] and lacunar infarcts [15]. However, the temporal association cannot be inferred from cross-sectional studies.

In the only prospective study reported, vitamin D concentrations were not associated with white matter hyperintensity progression, incident white matter hyperintensity score or incident infarcts in 888 healthy adults with a mean age of 62.3 years over a ten year follow-up period [20]. However, approximately 40% of participants were lost to follow-up, which might have biased the findings towards the null due to non-random attrition. To expand, low vitamin D concentrations could have led to the development of cerebrovascular abnormalities, which in turn resulted in participants dropping out before the follow-up scan.

We recently observed that low vitamin concentrations were associated with a substantially increased risk of all-cause dementia and AD in 1,658 elderly adults from the US-based Cardiovascular Health Study (CHS) [1]. In order to investigate the potential mechanisms underlying these associations we investigated whether vitamin D concentrations were associated with the risk of developing cerebrovascular and neurodegenerative neuroimaging abnormalities in the same population.

## Materials and Methods

### Participants

Participants were selected from the CHS, a large prospective population-based cohort in the US designed to investigate the underlying causes of cardiovascular disease in older men and women. Further details regarding the CHS have been published elsewhere [21]. The initial cohort consisted of 5,888 participants, of these 4,692 ambulatory participants had complete exam data in 1992–93. Serum 25-hydroxyvitamin D (25(OH)D) concentrations were not measured in 1,424 participants who had prevalent cardiovascular disease or stroke (one or more of the following: coronary heart disease, congestive heart failure, claudication, atrial fibrillation, pacemaker, implantable cardioverter-defibrillator, stroke or transient ischemic attack), determined by medical records, electrocardiograph findings and self-report [22]. Out of 3,268 participants, a further 945 were excluded as they had insufficient serum volumes for the vitamin D assay to be performed (<500 μl). Additional exclusions were made on the basis of missing adjudicated dementia status (n = 596) and prevalent dementia at baseline (n = 69). Of the 1,658 participants, for both MRI scans 1 and 2, 1,017 (61.3%) had complete white matter grade information, 994 (60.0%) had complete ventricular grade information and 1,108 had complete infarct information (66.8%). Those lost to follow-up (defined as participants with missing data for at least one of the three neuroimaging outcomes for the second MRI scan, N = 668) were more likely to be older (mean [SD], 74.6 [5.0] years vs 73.0 [4.0] years, *p*<0.001), had lower serum 25(OH)D concentrations (mean [SD], 63.3 [25.9] nmol/L vs 66.1 [26.8] nmol/L, *p* = 0.03), and were less educated (25.6% vs 19.8% did not finish high school, 54.2% vs 55.5% finished high school/some college/vocational qualifications, and 20.2% vs 24.7% completed college or professional qualifications, *p* = 0.007), but they were no more likely to be female (71.0% vs 68.1%, *p* = 0.21) or non-white (11.7% vs 12.9%, *p* = 0.45). In the main analyses, multiple imputation was used to restore the missing data for all neuroimaging outcomes resulting in a final sample size of 1,658. The following institutional review boards at each participating institution approved the research protocols; the University of California, Johns Hopkins University, Wake Forest University School of Medicine and University of Pittsburgh. All participants provided written informed consent during the course of the study.

### Serum 25(OH)D Measurement

Serum samples were collected once at baseline in 1992–93 and were stored at -70°C at the Laboratory for Clinical Biochemistry Research at the University of Vermont, and measurements were performed by the University of Washington Clinical Nutrition Research Unit in 2008 [23]. Total 25(OH)D (the sum of 25(OH)D_2_ and 25(OH)D_3_) was measured using liquid chromatography and tandem mass spectrometry (LC-MS) on a Waters Quattro micro mass spectrometer (Waters, Milford, Massachusetts); the inter-assay coefficient of variation was <3.4% [22]. Calibration of serum 25(OH)D concentrations were verified using SRM 972 from the National Institutes Standards and Technology [24]. Serum 25(OH)D was categorised using the following clinically relevant cut points: <25 nmol/L (severely deficient), ≥25 nmol/L to <50 nmol/L (deficient), ≥50 nmol/L (sufficient) [1].

### Neuroimaging Outcomes

Out of the original 5,888 participants, a total of 2,116 underwent both MRI scans, with the first scan conducted between 1991–1994 and the second scan conducted between 1997–1999. The MRI scans were performed on either General Electric or Picker 1.5 Tesla scanners at three field centres and on a 0.35 Tesla Toshiba instrument at one field center [25]. The scanning protocol involved a sagittal T_1_ weighted localizing scan, with subsequent axial spin density/T_2_ and T_1_ axial weighted scans aligned parallel to the anterior commissure-posterior commissure line [26]. Neuroradiologists who were unaware of clinical and demographic information estimated white matter and ventricular grades on a ten-point scale from 0–9 [25]. A higher grade indicates either more white matter disease, as defined by total extent of periventricular and subcortical white matter abnormalities, or enlarged ventricular volume [27]. In 2001–2002 white matter and ventricular grade were re-estimated with the baseline and follow-up scans read in a side-by-side fashion in order to improve rating reliability [28]. Infarcts were defined as either absent or present. The presence of infarcts was based on evidence of an area of abnormal signal intensity in a vascular distribution that lacked mass effect on the MRI scans [29]. All infarcts were ≥3mm in size as abnormalities <3mm could not be reliably detected. Change variables were derived for white matter and ventricular grade by subtracting the baseline scan grade from the follow-up scan grade. Due to the low number of participants who worsened by more than one grade for white matter (n = 45) and ventricular grade (n = 3), each variable was further categorised into two groups. The first group was defined as no change and the second group was defined as worsening grade by ≥1 between baseline and follow-up scans. 2 participants who had an improved white matter grade of one at follow-up and 6 participants who had an improved ventricular grade of one at follow-up were classified as no change. Similarly, a binary variable was derived for infarcts with the first group defined as no incident infarcts and the second group defined as developing incident infarcts.

### Covariates

We identified the following variables as potential confounders in the relationship between 25(OH)D concentrations and the development of neuroimaging abnormalities: age in years, season of blood collection (categories used for analysis—December-February; March-May; June-August; September-November), education status (did not finish high school; finished high school/some college/vocational qualifications; college/professional qualifications), sex, body mass index (BMI in kg/m^2^), smoking (non-smoker; current smoker), alcohol consumption (National Institute on Alcohol Abuse and Alcoholism definitions:- non-drinkers; moderate drinkers (women—≤7 drinks/week; men—≤14 drinks/week); heavy drinkers (women—>7 drinks/week; men—>14 drinks/week)), significant depressive symptoms (score ≥8 on the revised 10 item Center for Epidemiologic Studies Depression Scale [30]) and length of follow-up in years.

### Multiple Imputation

Multiple imputation by chained equations was used to account for the missing data observed in the neuroimaging outcomes as well as the covariates [31]. One participant was excluded from the imputed white matter model as they had a score of nine for the first scan, whilst 415 participants with prevalent infarcts were excluded from the imputed infarcts model. This resulted in a total of 1,658 participants for ventricular grade, 1,657 for white matter grade and 1,243 for infarcts. 100 imputations were generated for each variable with missing data. The following predictive variables were entered into the imputation model: age, sex, education, depressive symptoms, alcohol consumption, smoking, BMI, season of blood collection, ethnicity, hypertension, diabetes, kidney function, continuous change in 3MSE score per year from baseline to participants last follow-up, incident dementia, incident stroke, length of follow-up, baseline white matter grade, baseline ventricular grade and baseline infarcts. The imputation models were run separately for each neuroimaging outcome.

### Statistical Analysis

Multivariate logistic regression models were used to investigate the association between baseline serum 25(OH)D and the risk of developing incident neuroimaging abnormalities. Multiple imputation was performed prior to the main analyses. In basic models we adjusted for age, season of blood collection and length of follow-up. In fully adjusted models we controlled for the same covariates as the basic model as well as education, sex, BMI, smoking, alcohol consumption and depressive symptoms.

In secondary analyses, we repeated the main analyses by classifying serum 25(OH)D concentrations as a binary variable using the following cut-points: <50 nmol/L and ≥50 nmol/L. We also analysed serum 25(OH)D concentrations as a continuous variable. Continuous serum 25(OH)D concentrations were standardised to have a mean of 0 and a SD of 1 and were then normalised using a log transformation in order to address the positive skew.

In sensitivity analyses, the main analyses were rerun using propensity score weighting as opposed to multiple imputation to account for the missing data. This was to investigate whether either method of addressing missing data could bias the results. The variables used to derive the propensity score weights were the same as those used in the multiple imputation models. Additionally, the main analyses were repeated using only participants with complete data in order to explore whether either multiple imputation or propensity score weighting drastically alters the pattern of association. In the complete cases, 292 (28.7%) out of 1,017 developed worsening white matter grade, 275 (27.7%) out of 994 developed worsening ventricular grade and 153 (17.8%) out of 851 developed incident infarcts.

Multivariate logistic regression models were used to investigate the cross-sectional association between clinically relevant categories of serum 25(OH)D concentrations and prevalent white matter abnormalities (2 categories; grade <3 (N = 779) and grade ≥3 (N = 239)) [27], ventricular abnormalities (2 categories; grade <5 (N = 866) and grade ≥5 (N = 129)) [27] and infarcts (2 categories; present (N = 415) and absent (N = 1,237)).

P-values were two sided throughout with statistical significance preset at 0.05. Multiple imputation, propensity score weighting and all statistical analyses were performed using Stata/SE version 13 (StataCorp, College Station, Texas).

## Results

Table 1 presents the baseline characteristics for the total population. Compared to those with sufficient and deficient 25 (OH)D concentrations, participants with severe 25(OH)D deficiency tended to be older, have blood collected between December and May, be less educated, female, a smoker, have depressive symptoms, diabetes, treated hypertension and be of non-white ethnicity. There appeared to be little difference across categories of 25(OH)D concentrations in alcohol use and untreated hypertension. There was no difference in follow-up length between all categories of 25(OH)D concentrations.

**Table 1:** Baseline characteristics of 1,658 CHS Participants by serum 25(OH)D concentration.

		Serum 25(OH)D, nmol/L		
	All	≥50	≥25 to <50	<25
Characteristic	(n = 1,658)	(n = 1,169)	(n = 419)	(n = 70)
Age (y), *M*(SD)	73.6 (4.5)	73.6 (4.4)	73.7 (4.6)	74.1 (5.1)
Season tested, No. (%)				
Dec-Feb	363 (21.9)	207 (17.7)	133 (31.7)	23 (32.9)
Mar-May	383 (23.1)	207 (17.7)	144 (34.4)	32 (45.7)
Jun-Aug	475 (28.7)	407 (34.8)	60 (14.3)	8 (11.4)
Sep-Nov	437 (26.4)	348 (29.8)	82 (19.6)	7 (10.0)
Education (n = 1,655), No. (%)				
Did not finish high school	366 (22.1)	243 (20.8)	103 (24.7)	20 (28.6)
Finished high school/some college /vocational	910 (55.0)	644 (55.1)	226 (54.2)	40 (57.1)
College/professional	379 (22.9)	281 (24.1)	88 (21.1)	10 (14.3)
Female, No. (%)	1148 (69.2)	756 (64.7)	330 (78.8)	62 (88.6)
BMI (kg/m^2^), *M*(SD)	26.5 (4.5)	26.1 (4.2)	27.7 (5.1)	27.4 (5.3)
Current smoker (n = 1,615), No. (%)	149 (9.2)	93 (8.2)	46 (11.2)	10 (14.7)
Alcohol use (n = 1,656), No. (%)^a^				
Non-drinkers	898 (54.2)	613 (52.4)	245 (58.6)	40 (58.0)
Moderate drinkers	621 (37.5)	452 (38.7)	145 (34.7)	24 (34.8)
Heavy drinkers	137 (8.3)	104 (8.9)	28 (6.7)	5 (7.3)
Depressive symptoms (CES-D score ≥8), No. (%)	359 (21.7)	221 (18.9)	117 (27.9)	21 (30.0)
Diabetes, No. (%)^b^	175 (10.6)	95 (8.1)	64 (15.3)	16 (22.9)
Hypertension, No. (%)				
Normal	696 (42.0)	526 (45.0)	151 (36.0)	19 (27.1)
Treated	639 (38.5)	412 (35.2)	189 (45.1)	38 (54.3)
Untreated	323 (19.5)	231 (19.8)	79 (18.9)	13 (18.6)
Years of follow-up (n = 1,087), *M*(SD)	5.0 (0.6)	5.0 (0.6)	5.0 (0.6)	5.0 (0.6)
White, No. (%)	1452 (87.6)	1088 (93.1)	322 (76.9)	42 (60.0)
White matter grade (n = 1,018), *M*(SD)	1.7 (1.4)	1.7 (1.4)	1.6 (1.3)	2.0 (1.4)
Ventricular grade (n = 995), *M*(SD)	3.1 (1.2)	3.2 (1.2)	2.9 (1.2)	3.3 (1.4)
Infarcts (n = 1,652), No. (%)	415 (25.1)	289 (24.8)	100 (23.9)	26 (37.7)

Abbreviations: 25(OH)D, 25-hydroxyvitamin D; BMI, body mass index, CES-D, Center for Epidemiologic Studies Depression Scale[30] (revised 10 item scale)

^a^ National Institute on Alcohol Abuse and Alcoholism guidelines

^b^ American Diabetic Association guidelines

Participants with complete MRI data for any neuroimaging outcome were followed up for a mean of 5.0 years (N = 1,087), SD 0.6, median 5.0, range 3.2–7.5). In both minimally and fully adjusted models, there was no significant association observed between clinically relevant categories of serum 25(OH)D and the risk of worsening white matter grade, worsening ventricular grade or incident infarcts (Table 2). Similar findings were observed when entering serum 25(OH)D in the models as a binary as opposed to a three group categorical variable (Table 3). Furthermore, there was no significant association observed between log-transformed standardised 25(OH)D concentrations and either worsening white matter grade (Odds Ratio [OR] = 1.04, 95% CI: 0.90–1.21, *p =* 0.59), worsening ventricular grade (OR = 1.05, 95% CI: 0.90–1.23, *p =* 0.53) or incident infarcts (OR = 1.08, 95% CI: 0.89–1.30, *p* = 0.45) in multivariate adjusted logistic regression models.

**Table 2:** Odds ratio of developing neuroimaging abnormalities by 3 categories of serum 25(OH)D concentrations after multiple imputation.

		Serum 25(OH)D, nmol/L			
		≥50	≥25 to <50	<25	
Neuroimaging Outcome	No. of participants	OR	OR (95% CI)	OR (95% CI)	*p*-value for linear trend
White matter grade					
Model A^a^	1,657	1 (Reference)	1.09 (0.78–1.53)	0.76 (0.36–1.64)	0.94
Model B^b^	1,657	1 (Reference)	1.09 (0.76–1.55)	0.76 (0.35–1.66)	0.92
Ventricular grade					
Model A^a^	1,658	1 (Reference)	1.15 (0.83–1.60)	0.51 (0.21–1.22)	0.69
Model B^b^	1,658	1 (Reference)	1.12 (0.79–1.59)	0.49 (0.20–1.19)	0.56
Infarcts					
Model A^a^	1,243	1 (Reference)	0.74 (0.49–1.13)	2.00 (0.86–4.61)	0.78
Model B^b^	1,243	1 (Reference)	0.73 (0.47–1.13)	1.95 (0.84–4.54)	0.82

Abbreviations; OR, Odds Ratio; CI, Confidence Interval

^a^ Adjusted for age, season of vitamin D collection and length of follow-up

^b^ Adjusted for Model A and education, sex, BMI, smoking, alcohol consumption and depressive symptoms

**Table 3:** Odds ratio of developing neuroimaging abnormalities by 2 categories of serum 25(OH)D concentrations after multiple imputation.

		Serum 25(OH)D, nmol/L		
		≥50	<50	
Neuroimaging Outcome	No. of participants	OR	OR (95% CI)	*p*-value
White matter grade				
Model A^a^	1,657	1 (Reference)	1.03 (0.74–1.44)	0.84
Model B^b^	1,657	1 (Reference)	1.03 (0.73–1.46)	0.85
Ventricular grade				
Model A^a^	1,658	1 (Reference)	1.05 (0.75–1.46)	0.79
Model B^b^	1,658	1 (Reference)	1.01 (0.72–1.42)	0.95
Infarcts				
Model A^a^	1,243	1 (Reference)	0.86 (0.57–1.30)	0.46
Model B^b^	1,243	1 (Reference)	0.86 (0.56–1.31)	0.48

Abbreviations; OR, Odds Ratio; CI, Confidence Interval

^a^ Adjusted for age, season of vitamin D collection and length of follow-up

^b^ Adjusted for Model A and education, sex, BMI, smoking, alcohol consumption and depressive symptoms

In sensitivity analyses, using propensity score weighting as opposed to multiple imputation to account for missing data produced similar findings to the main analyses (Table 4). Additionally, the observed associations between serum 25(OH)D concentrations and neuroimaging abnormalities remained similar in participants with complete MRI data (Table 5).

**Table 4:** Odds ratio of developing neuroimaging abnormalities by 3 categories of serum 25(OH)D concentrations after propensity score weighting.

		Serum 25(OH)D, nmol/L			
		≥50	≥25 to <50	<25	
Neuroimaging Outcome	No. of participants	OR	OR (95% CI)	OR (95% CI)	*p*-value for linear trend
White matter grade					
Model A^a^	987	1 (Reference)	1.12 (0.77–1.63)	0.77 (0.34–1.75)	0.95
Model B^b^	987	1 (Reference)	1.12 (0.77–1.64)	0.75 (0.34–1.69)	0.99
Ventricular grade					
Model A^a^	964	1 (Reference)	1.13 (0.78–1.63)	0.56 (0.23–1.38)	0.75
Model B^b^	964	1 (Reference)	1.07 (0.74–1.55)	0.51 (0.21–1.26)	0.49
Infarcts					
Model A^a^	824	1 (Reference)	0.70 (0.43–1.13)	1.70 (0.70–4.11)	0.69
Model B^b^	824	1 (Reference)	0.70 (0.42–1.15)	1.76 (0.72–4.29)	0.74

Abbreviations; OR, Odds Ratio; CI, Confidence Interval

^a^ Adjusted for age, season of vitamin D collection and length of follow-up

^b^ Adjusted for Model A and education, sex, BMI, smoking, alcohol consumption and depressive symptoms

**Table 5:** Odds ratio of developing neuroimaging abnormalities by 3 categories of serum 25(OH)D concentrations in participants with complete MRI data.

		Serum 25(OH)D, nmol/L			
		≥50	≥25 to <50	<25	
Neuroimaging Outcome	No. of participants	OR	OR (95% CI)	OR (95% CI)	*p*-value for linear trend
White matter grade					
Model A^a^	1,017	1 (Reference)	1.02 (0.72–1.45)	0.72 (0.33–1.55)	0.65
Model B^b^	988	1 (Reference)	1.04 (0.72–1.49)	0.80 (0.36–1.76)	0.82
Ventricular grade					
Model A^a^	994	1 (Reference)	1.11 (0.79–1.58)	0.48 (0.19–1.17)	0.52
Model B^b^	965	1 (Reference)	1.06 (0.74–1.52)	0.47 (0.19–1.16)	0.40
Infarcts					
Model A^a^	851	1 (Reference)	0.71 (0.44–1.12)	1.76 (0.77–4.03)	0.86
Model B^b^	825	1 (Reference)	0.69 (0.43–1.12)	1.73 (0.71–4.19)	0.74

Abbreviations; OR, Odds Ratio; CI, Confidence Interval

^a^ Adjusted for age, season of vitamin D collection and length of follow-up

^b^ Adjusted for Model A and education, sex, BMI, smoking, alcohol consumption and depressive symptoms

In cross-sectional analyses, the odds of prevalent infarcts were 91% higher in those with severe 25(OH)D deficiency compared to those with sufficient 25(OH)D concentrations in the fully adjusted model (OR = 1.91, 95% CI 1.12–3.27) (Table 6). However there were no differences in the odds of prevalent infarcts in those with 25(OH)D deficiency compared to those sufficient and the linear trend across all three categories was not significant (p = 0.15). Similarly, there were no significant associations between the odds of prevalent white matter or ventricular abnormalities and serum 25(OH)D concentrations.

**Table 6:** Odds ratio of prevalent neuroimaging abnormalities by 3 categories of serum 25(OH)D concentrations in participants with complete MRI data at baseline.

		Serum 25(OH)D, nmol/L			
		≥50	≥25 to <50	<25	
Neuroimaging Outcome	No. of participants	OR	OR (95% CI)	OR (95% CI)	*p*-value for linear trend
White matter grade					
Model A^a^	1,018	1 (Reference)	0.75 (0.51–1.10)	1.41 (0.70–2.85)	0.75
Model B^b^	989	1 (Reference)	0.76 (0.51–1.13)	1.29 (0.62–2.70)	0.65
Ventricular grade					
Model A^a^	995	1 (Reference)	0.63 (0.38–1.04)	1.34 (0.58–3.10)	0.48
Model B^b^	966	1 (Reference)	0.71 (0.41–1.20)	1.49 (0.60–3.66)	0.78
Infarcts					
Model A^a^	1,652	1 (Reference)	1.02 (0.78–1.34)	1.98 (1.18–3.34)	0.08
Model B^b^	1,609	1 (Reference)	0.98 (0.74–1.30)	1.91 (1.12–3.27)	0.15

Abbreviations; OR, Odds Ratio; CI, Confidence Interval

^a^ Adjusted for age, season of vitamin D collection and length of follow-up

^b^ Adjusted for Model A and education, sex, BMI, smoking, alcohol consumption and depressive symptoms

## Discussion

In this large US population-based prospective cohort study, after adjusting for a wide range of potential confounders, we found no association between serum 25(OH)D concentrations and the risk of developing incident neurodegenerative or cerebrovascular abnormalities in elderly adults free from prevalent dementia, stroke and cardiovascular disease. The lack of association was consistent when accounting for missing data through multiple imputation and propensity score weighting, as well as restricting the analyses to complete cases. Severe 25(OH)D deficiency was associated with an increased risk of prevalent infarcts compared to those with 25(OH)D sufficiency; however there were no further associations between 25(OH)D concentrations and prevalent neuroimaging abnormalities.

The only previous prospective study in the area did not observe any association between vitamin D concentrations and the development of white matter hyperintensities and incident infarcts [20]. However, the population consisted of younger adults with a mean age of 62.3 years as well as a high rate of missingness at the follow-up scan, both of which might have restricted the possibility of observing significant associations [20]. We addressed these limitations and also found no association with the development of white matter hyperintensities or incident infarcts, additionally we did not observe an association with increased ventricular atrophy.

These findings are in contrast with the majority of cross-sectional neuroimaging studies, which have largely found consistent associations between lower vitamin D concentrations and an increased risk of cerebrovascular and neurodegenerative abnormalities. Six cross-sectional studies found that low vitamin D concentrations were significantly associated with more white matter abnormalities [4,10,12,13,15,17], although three studies found no association with white matter abnormalities or volume [6,8,19]. Whilst, one study found that vitamin D deficiency (≤50 nmol/L) was associated with a 28% increase in lateral ventricle volume compared to those with vitamin D sufficiency (>50 nmol/L) [8].

We found that severe 25(OH)D deficiency (<25 nmol/L) was associated with an increased prevalence of infarcts compared to those with 25(OH)D sufficiency (≥50 nmol/L). This association was also similar in the prospective analyses for incident infarcts, however it did not reach statistical significance. There have been several studies that have implicated low vitamin D concentrations in the development of infarcts. Adult rats treated with vitamin D_3_ via injection for a period of eight days demonstrated a significant reduction in middle cerebral arterial litigation-induced cortical infarction in the cortex compared to control rats injected with saline [32]. Furthermore, in a meta-analysis consisting of 26,596 participants, low 25(OH)D concentrations were prospectively associated with an increased risk of ischemic stroke [33]. In cross-sectional neuroimaging studies, lower vitamin D concentrations are associated with increased prevalence of large vessel infarcts [4], lacunar infarcts [15] as well as larger ischemic infarct volume [18], but not small vessel infarcts [4]. It is possible that the small sample size in the current study was too underpowered to detect a statistically significant association with incident infarcts in those with severe 25(OH)D deficiency.

Our study has several strengths. It is the first large prospective study to investigate the association between 25(OH)D concentrations and the risk of developing neuroimaging abnormalities in ambulatory elderly adults. We also utilised multiple imputation and propensity score weighting to address attrition during follow-up. Scans one and two were assessed in a side-by-side fashion for white matter and ventricular grade which improved rating reliability as opposed to assessing the scans separately. In the current population, we previously identified a strong association between low vitamin D concentrations and the risk of incident all-cause dementia and AD [1] whilst the MRI scans were incorporated in the diagnosis of these conditions [34]. Therefore, this population is well-suited to explore the potential cerebrovascular and neurodegenerative mechanisms that underlie these observed associations using neuroimaging technology. Our study also has several limitations. 25(OH)D concentrations were only measured in participants free from prevalent cardiovascular disease and stroke which might have reduced the likelihood of them developing cerebrovascular abnormalities at follow-up. This is supported by the low number of incident vascular dementia cases (N = 15) found in the population. However, of the complete cases, 28.7% developed worsening white matter grade and 18% developed incident infarcts, which should be sufficient to detect an association. The outcomes were assessed by two neuroradiologists, which is likely to have introduced a degree of subjective bias and human error, whereas quantitative software tools would have provided higher degree of precision. Similarly it would have been useful to have quantitative measures of white matter and ventricular volume as opposed to relying on a 10-point scale score. Length of follow-up ranged from 3.2 to 7.5 years, so it is possible that those who were scanned within the lower range did not have an adequate amount of time to develop neuroimaging abnormalities. Despite using methods to address the issue of missing data at follow-up it is possible that this was not sufficient to account for the possibility of non-random attrition. It is also possible that measurement error could have been introduced when assessing the neuroimaging outcomes at two different time points, although this is unlikely to have resulted in systematic bias.

In elderly US-based adults free from prevalent dementia, stroke and cardiovascular disease we found that vitamin D concentrations at baseline were not prospectively associated with the risk of developing worsening white matter grade, worsening ventricular grade or incident infarcts. However, there was a non-significant tendency towards an increased risk of incident infarcts in those with severe 25(OH)D deficiency compared to those sufficient. Given the limitations, such as a high proportion of participants lost to follow-up, further prospective neuroimaging studies incorporating a wider range of cerebrovascular and neurodegenerative abnormalities measured with greater precision are warranted.
